# Annotations

**Published:** 1901-11-30

**Authors:** 


### ANNOTATIONS.

### Rate-Supported Sanatoria.

The powers possessed by sanitary authorities of building hospitals for the reception of other than infectious diseases have been so little exercised that many people are hardly aware that they exist. Such is the fact, however, for although, curiously enough, local authorities cannot spend sixpence in treating the sick in their own homes, they may spend thousands of pounds in building hospitals for their reception, quite irrespective of the disease from which they suffer. Not only are the rates at their disposal for the building of hospitals, but any sanitary authority which is anxious to distinguish itself in liberality need not wait to build a hospital of its own, but may "contract for the use of any such hospital or part of a hospital or place of reception; or enter into any agreement with any person having the management of any hospital, for the reception of the sick inhabitants of their district on payment of such annual or other sum as may be agreed on." This is wide enough in all conscience, and there would seem nothing to prevent any local sanitary authority from proceeding under this section of the Public Health Act, either to set up a general hospital of its own or to subscribe "such annual or other sum as may be agreed on" to any existing hospital for the reception of "the sick inhabitants of their district." So far as the law is concerned the "municipalisation of the hospitals" has long been *un fait accompli.* Yet, although this clause is a quarter of a century old, it has remained practically a dead letter. Under the influence of popular movements, however, old legal machinery is apt to be furbished up, and the sanitary authorities of Hampshire, stimulated by a resolution passed at the recent Tuberculosis Congress to the effect "that the provision of sanatoria is an indispensable part of the measures necessary for the diminution of tuberculosis," are now being urged to spend their money in providing such sanatoria. Which is all very well, and sounds very nice. It must, however, be recognised that it is the thin end of a very big wedge, and that if a sanitary authority can build a sanatorium for the tuberculous, it can just as well build a hospital for those who are "sick" from any other form of disease, and that it will soon be asked to do. This is a large order, and under the circumstances it may be well to step warily.

### Kissing the Baby.

That kissing is an instinct we do not believe, but it has become so engrained as a habit that some people fly to it on the slightest temptation, and actually seem to enjoy it. It would then be idle to attempt, in this generation at least, to put a stop to such a proceeding, and so far as it affects people who have arrived at years of discretion and have a say in the matter, it may go on for aught we care. The kissing of helpless children, however, is another affair. There can be but little doubt that by this stupid custom infection is often conveyed even from mother to child, and frequently from nursemaid to infant. But the evil extends much further, for the training which these much-beslavered infants receive during the first years of their lives sticks to them, and when they go to school leads to endless troubles. These wretched little children are brought up to kiss whatever they love—a purely conventional mode of expression—and so they kiss each other. Poor little brats, receiving in return for their affectionate embrace infections which too often kill them! It is too bad. We do not say that a mother should not kiss her own child. Her baby is part of herself, and we suppose she must be allowed to do as she likes with it, although even a mother ought to bear in mind that her colds and sore throats are distinctly catching. But for strangers to kiss a baby is a piece of abominable impertinence. The whole business ought to be put a stop to. Think of the horrid moment when the adored baby toddles forward with its mouth up waiting to be kissed! And so far does folly go that most mothers would actually be offended if the visitor failed to respond to baby's "sweet advances"—very sweet—sometimes absolutely sticky! The fact is that kissing is the very antithesis of sanitary decency. To "engaged" young people it may perhaps be permitted as a foretaste of that more complete union which they hope to arrive at. But among others it ought to be regarded as an outrage, and to impose such a rite on helpless children and thus to teach them to pass on their infections to those of their infantile companions whom they most love should be regarded as an unforgivable sanitary sin.

### The Army Medical Service.

The appointment of the members of the Advisory Board, in accordance with the new scheme for the governance of the Army Medical Service, appears to indicate that this scheme, at any rate in its general outline, is to be acted upon. It seems desirable then before any final decision as to details is arrived at to urge the importance of reconsidering the clause which imposes an examinational test upon officers after 18 years of service. In the junior ranks examinations are all very well, but when men have been knocking about the world for 18 years, the most futile of all tests of merit is an examination, while the imposition of such a test is sure to act as a most potent means of deterring men from entering the service. Whatever may be the attractions offered by the army to those who join it only for a few years, the real solid attraction by which good men are drawn to it as their main work in life and by which they are tempted to remain in it notwithstanding all the rebuffs and hardships which they encounter, is the right to retire with a pension of £1 a day at the end of twenty years of service. Much has been said of the hardships, the deprivation of leave, the rank injustice in regard to pay in India, and the many snubs which the medical service has received, and people ask, why do men stay in such a service? The answer is the pension. This is the great attraction, this is the bait by which good men are drawn to and kept in the service; and we cannot but feel that to remove the certainty of a pension, and to make even continuance in the service depend upon success in passing an examination, the nature and the standard of which no one can guess at 18 years beforehand, is a very serious step, and one which is sure to add greatly to the difficulties of the Government in obtaining good officers for the Army Medical Service.

